# Newly Designed Human-Like Collagen to Maximize Sensitive Release of BMP-2 for Remarkable Repairing of Bone Defects

**DOI:** 10.3390/biom9090450

**Published:** 2019-09-04

**Authors:** Zhuoyue Chen, Zhen Zhang, Xiaoxuan Ma, Zhiguang Duan, Junfeng Hui, Chenhui Zhu, Donggang Zhang, Daidi Fan, Lijun Shang, Fulin Chen

**Affiliations:** 1 Provincial Key Laboratory of Biotechnology of Shaanxi, Northwest University, 229 TaiBai North Road, Xi’an 710069, China; 2 Key Laboratory of Resource Biology and Modern Biotechnology in Western China, Ministry of Education, Northwest University, 229 TaiBai North Road, Xi’an 710069, China; 3 Shaanxi Key Laboratory of Degradable Biomedical Materials, School of Chemical Engineering, Northwest University, 229 TaiBai North Road, Xi’an 710069, China; 4 Shaanxi R&D Center of Biomaterial and Fermentation Engineering, School of Chemical Engineering, Northwest University, 229 TaiBai North Road, Xi’an 710069, China; 5 Yantai Zhenghai Biotechnology Co, Ltd., 10 Hengshan Road, Yantai Economic & Technological Development Area, Yantai 264000, China; 6 School of Chemistry and Biosciences, Faculty of Life Sciences, University of Bradford, Bradford BD7 1DP, UK

**Keywords:** human-like collagen, bone morphogenetic protein-2, osteoinduction, bone overgrowth, sensitive release system

## Abstract

Designing the “ideal” hydrogel/matrix which can load bone morphogenetic protein-2 (BMP-2) in a low dose and with a sustained release is the key for its successful therapeutic application to enhance osteogenesis. The current use of natural collagen sponges as hydrogel/matrix is limited due to the collagen matrix showing weak mechanical strength and unmanageable biodegradability. Furthermore, the efficiency and safe dose usage of the BMP-2 has never been seriously considered other than purely chasing the lowest dose usage and extended-release time. In this paper, we customized a novel enzymatically cross-linked recombinant human-like collagen (HLC) sponge with low immunogenicity, little risk from hidden viruses, and easy production. We obtained a unique vertical pore structure and the porosity of the HLC, which are beneficial for Mesenchymal stem cells (MSCs) migration into the HLC sponge and angiopoiesis. This HLC sponge loading with low dose BMP-2 (1 µg) possessed high mechanical strength along with a burst and a sustained release profile. These merits overcome previous limitations of HLC in bone repair and are safer and more sensitive than commercial collagens. For the first time, we identified that a 5 µg dose of BMP-2 can bring about the side effect of bone overgrowth through this sensitive delivery system. Osteoinduction of the HLC-BMP sponges was proved by an in vivo mouse ectopic bone model and a rat cranial defect repair model. The method and the HLC-BMP sponge have the potential to release other growth factors and aid other tissue regeneration. Additionally, the ability to mass-produce HLC in our study overcomes the current supply shortage, which limits bone repair in the clinic.

## 1. Introduction

Treatment for serious fractures and critical size bone defects (defects that do not heal without intervention) always requires the assistance of osteoinductive agent, such as bone morphogenetic proteins (BMPs) [1]. BMP-2 is one of the most potent osteoinductive agents in BMPs family [2,3], and its successful use in bone therapy and treatments has recently been reported [1]. Currently, BMP-2 is used mainly in dental treatment, open tibial fractures, cartilage damage, cancer, and spinal surgery to enhance and improve bone therapy [1]. BMP-2 can now be produced by using recombinant human DNA technology (rhBMP-2), but it can only initiate bone formation in solution form. This makes it difficult for BMP-2 to be retained at the injured site for enough time to help regeneration of the bone [4,5,6,7]. Therefore, a carrier system which can load with BMP-2 in an appropriately low dose and with a sustained release profile is urgently needed. The most commonly used BMP-2 carrier materials include ceramics [8,9], synthetic polymers [10,11], natural polymers (such as collagen, chitosan, silk fibroin, alginate, gelatin) [12,13,14,15], and their composites [16,17]. Among these carrier materials, collagen has some advantages. For example, collagen is one of the main components of the extracellular matrix of bone tissue, and natural collagen solutions can convert to collagen sponges after lyophilization to provide the structural matrix for bone tissue regeneration, and this can avoid the complex shaping processes [18]. Collagen sponges can gradually release the proteins and can be easily molded into the defected area, easily infiltrated by the cells responsible for bone formation and readily transformed into bone, making it an ideal carrier for rhBMP-2 [19]. Meanwhile, collagens have high binding affinity to BMPs [20], and this property makes them even more widely exploited in bone repair and regeneration [21]. An economic analysis reported that the use of rhBMP-2 with collagen would result in savings of $ 3183 to $ 5697 per patient due to faster healing rates and thus reducing sickness-associated payouts [22].

But there are several areas which still need to be optimized and refined on BMP-2, collagen, and the combined use of both. For example, most commercial collagens are from animals, and their applications are restricted due to their potential for immunogenicity [23] and virus contamination [24]. They also lack mechanical strength and have unpredictable biodegradability [7]. In addition, using commercial collagens to release the BMP-2 normally results in a very low utilization rate of the BMP-2 [25]. Therefore, designing the “ideal” hydrogel/matrix which can load BMP-2 in a low dose and with a sustained release is crucial for its successful therapeutic application to enhance osteogenesis. This includes choosing the source and dose of BMP-2 to be used, controlling the timing for BMP-2 to form hydrogel/matrix with the carrier system (i.e., Collagen), and designing and forming the favorable structure of the loading matrix of BMP-2, etc. Current use of natural collagen sponges as hydrogel/matrices is limited due to the collagen matrix showing weak mechanical strength and unmanageable biodegradability.

Full-length recombinant collagens were, therefore, developed to overcome the above disadvantages [26,27,28]. They are similar in structure to human collagen but have good biocompatibility, such as low immunogenicity, and have little risk from hidden viruses. However, their weak mechanical strength limits their applications mainly to neural, skin, and cardiac therapy [29,30,31]. Furthermore, their lack of stability and quick degradation during the purification process results in low-yields and high production costs, and consequently limits their applications, thus excluding them from bone and cartilage tissue engineering.

Some groups are studying recombinant human-like collagen (HLC) [32,33,34,35,36]. The backbone of this HLC is not a full-length human collagen gene code but is a novel sequence from several modified repeat sequences of the cDNA fragment transcribed from the mRNA with human collagen coding [32]. This repeat peptide sequence, (Gly-Xaa-Yaa)_n_ (where Xaa and Yaa represent one random amino acid) and the sequence template can be modified (change Xaa and Yaa) to suit cell residence and growth [32,33,34]. As far as we are aware that, apart from our group, there are only two groups (Tang et al. [35] and Yang et al. [36]) which have recently successfully expressed HLC (Table S1). However, compared with our group, apart from the differences in host *E. coli*, the expression of HLC was achieved through a shaking flask culture only in Yang et al.’s work [36]; and the yield of HLC was only 260 mg/L in a 10 L bench-top fermenter in Tang et al.’s study [35]. We first created recombinant human-like collagen (HLC, Patent No: ZL01106757.8) as early as 2002 [32] and then have continuously developed and optimized several recombinant HLCs since. In the early stage, the yield of HLC was too low and made it impossible to investigate its application. Nowadays, improved yield of the HLC can achieve 14 g/L in a 500 L fermenter at a relatively lower cost. This higher yield of the HLC has facilitated its wide applications in hemostatic sponge [34], skin injury treatments [37], and tissue engineering vascular scaffolds [38,39] in our group. However, its applications in the treatment of hard tissue injury are still very difficult due to its weak mechanical strength. In our previous studies, we used several cross-link agents, such as genipin, glutaraldehyde (GA), tyrosinase, and glutamine transaminase (TG) to improve the mechanical properties of the HLCs. We found that they all can make HLCs stronger, but the genipin and GA elicit either cytotoxic side-effects or immunological responses from the host [40,41]. The enzymes, including tyrosinase and TG, are otherwise generally non-toxic and non-immunogenic. However, compared with tyrosinase, the hydrogel cross-linked by TG is mechanically stronger and more stable. Meanwhile, other studies show that the porosity and pore size of the collagen sponges can be controlled through the “gradient freeze-drying” method [42] and combining with a TG cross-linker.

Based on our previous studies, in this paper, we designed a new HLC and used TG to enzymatically cross-link this HLC to control its mechanical strength and vertical pore structure. We then used this specially structured HLC sponge loaded with a small amount of BMP-2, 1 µg (HLC-BMP delivery system), to achieve a short burst release of BMP-2 followed by a steady slow process. To understand the mechanism connecting the HLC-BMP sponge and the cells, the physical and chemical properties of the HLC and HLC-BMP were analyzed by sodium dodecyl sulfate-polyacrylamide gel electrophoresis (SDS-PAGE), digital microscopy, scanning electronic microscopy (SEM), biomechanical analyzer and microplate reader. The biological properties of HLC-BMP (using HLC as the control) were also evaluated using in vitro Mesenchymal stem cells (MSCs) cultures. Furthermore, we used a mouse subcutaneous model and a rat cranial defect repair model to verify the osteoinduction of HLC-BMP sponges.

## 2. Materials and Methods

**Figure 1:**
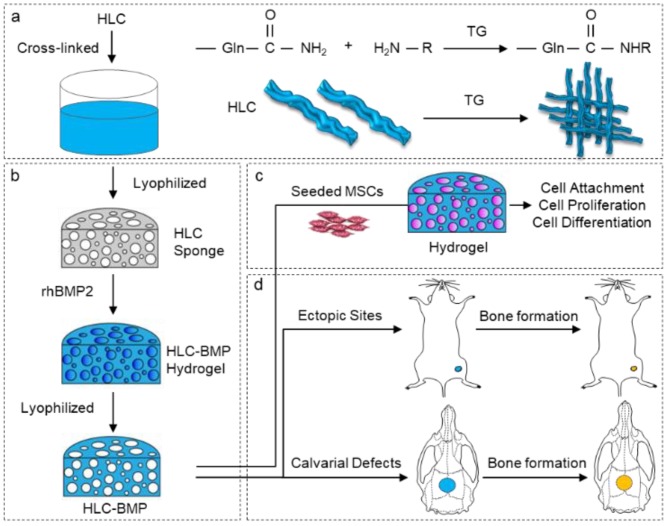
Schematic diagram of the process of enzymatically crosslink for the preparation of the human-like collagen-bone morphogenetic protein (HLC-BMP) sponge, and in vivo and in vitro tests. (**a**) HLC was enzymatically cross-linked, (**b**) Preparation of HLC-BMP sponge, (**c**) The in vitro test of the HLC-BMP with MSCs, (**d**) The in vivo efficacy test of the HLC-BMP.

The experimental design is shown in Figure 1. The pure HLC was enzymatically cross-linked by TG to form a pure HLC sponge scaffold. The detailed enzymatically crosslink process is shown in Figure 1a. As shown in Figure 1b, the cross-linked HLC hydrogel became an HLC sponge through lyophilization at the first step. After absorbed BMP-2, the HLC sponge became an HLC-BMP hydrogel. To obtain an HLC-BMP sponge, the HLC-BMP hydrogel was lyophilized in the end. After the physical and chemical properties of the HLC and HLC-BMP were analyzed, the biological properties of the HLC-BMP sponge (HLC as the control) were evaluated using in vitro cell cultures with MSCs (Figure 1c) and in vivo animal implantation (Figure 1d).

### 2.1. Fabrication of HLC-BMP2 Sponge

The recombinant *E. coli* BL21 had a plasmid with the HLC, kanamycin resistance, and temperature induction genes. Fed-batch cultivation of recombinant *E. coli* BL21 was carried out at 34 °C for 24 h in a fermenter (volume 500 L, Shanghai Baoxing Co. China, BIOTECH-500JS, Shanghai, China). Then a series of purification was carried out to obtain the HLC. The HLC is a macromolecular water-soluble protein, and normally its protein molecular weight and purity can be confirmed by SDS-PAGE. Then the water-soluble HLCs were cross-linked with TG. In brief, HLCs were dissolved in pseudo-physiological solution (PBS) buffer (pH = 6) to prepare a 10% (*w*/*v*) solution, and TG (20 U TG in 1 g HLC) was added immediately, and then the solution was thoroughly mixed. The solution was then kept at 4 °C for 48 h, and the prepared gels were lyophilized and stored at 4 °C. The recombinant human BMP-2 (rhBMP-2, Zhenghai Group Co. Ltd., Yantai, China, China Patent number CN 1807458A/1807459A; USA PCT: US8802396B2; European EP1970382B1) was reconstituted and diluted in buffer solution to concentrations of 10, 20, 30, 40, and 50 ng/µL, and each of the HLC sponges were soaked in 100 µL of the prepared rhBMP-2. As a result, 1, 2, 3, 4, and 5 µg of rhBMP-2 was loaded onto each of the HLC sponges, respectively.

### 2.2. Morphology Characterization of Cross-Linked HLC Sponge

The HLC sponges were checked with a digital microscope (VHX-5000, Keyence, Osaka, Japan) at 30× magnification. After being sputter-coated with gold, the microstructures and surface morphologies of the HLC sponges were then observed by scanning electronic microscopy (SEM, Hitachi S-4700, Tokyo, Japan).

### 2.3. Biomechanical Evaluation of HLC/HLC-BMP Sponges

A biomechanical analyzer (Instron-5542, Canton, MA, USA) was employed to evaluate the biomechanical properties. The HLC and HLC-BMP sponges were prepared in the form of cubes (15 mm in length, 7 mm in wide, and 15 mm in height). In brief, 80% of maximal deformation was continuously checked under 0.5 mm/min compressive strain to generate a stress-strain curve. The slope of this curve represented Young’s modulus of the HLC and HLC-BMP sponges illustrated in our previous study [10]. All tests were repeated four times.

### 2.4. Kinetics of Releasing rhBMP-2 from the HLC-BMP Hydrogel

The kinetics of releasing rhBMP-2 from the HLC-BMP sponge was measured to understand the interaction between HLC and rhBMP-2. First, the HLC sponges were soaked in the prepared rhBMP-2 to obtain the HLC-BMP hydrogel. Then the HLC-BMP hydrogel was lyophilized to form the HLC-BMP sponge. Finally, the release rate of rhBMP-2 from the HLC-BMP sponge was obtained using our previous method [10]. In brief, the HLC-BMP sponges were immersed into 5 mL PBS contained in a six-well plate and incubated (37 °C, 5% CO_2_, 95% relative humidity) for 28 days. At 1, 2, 3, 5, 7, 14, 21, and 28 days, the total release of rhBMP-2 was checked by ELISA according to the manufacturer’s protocol (Human BMP2 ELISA Kit, Abcam, ab119581, Lot GR3174552-1, Cambridge, MA, USA). All tests were repeated four times. To confirm that the rest of the rhBMP-2 remained in the scaffold, the HLC-BMP hydrogels were analyzed using biotinylated anti-human BMP-2 antibody from the Human BMP2 ELISA Kit (Abcam, ab119581, Lot GR3174552-1) and streptavidin-FITC (Sigma, Lot 117M4102V, Sigma-Aldrich, St. Louis, MO, USA) at days 1, 5, 14, and 28, respectively. The fluorescence images from the stained samples were obtained using a confocal laser scanning microscope (FV1000, Olympus Corporation, Tokyo, Japan).

### 2.5. In Vitro Studies

#### 2.5.1. Isolation and Culture of Rat MSCs

Sprague Dawley (SD) rats (6~8 weeks old and weight 220~250 g) were obtained from the animal holding unit of Northwest University, and samples of bone marrow were harvested in accordance with Institutional Animal Care and Use Committee (IACUC, ACUC2013015) approval of Northwest University. Rat MSCs were isolated and cultured using our previous method [43,44]. Briefly, the obtained marrow was suspended and cultured in DMEM containing 10% FBS. The medium was refreshed after 24 h to remove non-adherent cells. The medium was then changed every 3 days. Before the cells formed a confluent monolayer, they were digested using trypsin 0.25%, and cells at passage 2 were used for experiments. The cell density was adjusted to 5 × 10^7^ cells/mL with the medium before cell seeding.

#### 2.5.2. MSCs Attachment and Proliferation in the HLC/HLC-BMP Sponges

To investigate the interaction between the HLC/HLC-BMP sponges and the MSCs, MSCs attachment, and proliferation on the corresponding sponges were examined. First, the sterilized HLC and HLC-BMP sponges (cylinder of diameter and height: 8 × 3 mm) were placed into a 6-well plate. 1 × 10^6^ MSCs were seeded into each sponge and incubated for 4 h for them to attach. The specimens were then rinsed with PBS, fixed with 2.5% glutaraldehyde and followed by dehydrating for about 2 min in the gradient ethanol solutions from 30%, 50%, 70%, 90% to 100% for each concentration. After critical point drying, the specimens were eventually coated with gold-palladium. They were then examined by SEM.

The proliferation of the MSCs in the HLC/HLC-BMP sponge was separately determined using a CCK-8 kit according to the method described in our previous study [10]. Briefly, 4 × 10^3^ MSCs were seeded into each sponge. The number of cells in the sponge was determined using a CCK-8 kit at days 1, 2, and 3 according to the manufacturer’s protocol after the specimens had been washed by PBS. All experiments were repeated four times.

At day 3, the specimens were fixed in 4% paraformaldehyde (*v*/*v*) and stained with β-actin primary antibody (Host: Rabbit, Abcam, ab8227), donkey anti-rabbit secondary antibody (Alexa Fluor^®^ 594-conjugated, Jackson, 715-585-152, West Grove, PA, USA), followed by 4′,6-diamidino-2-phenylindole (DAPI). The specimens were then imaged using confocal microscopy (FV1000, Olympus Corporation, Tokyo, Japan), followed by analysis by the FV10-ASW software (Olympus Co. Japan). Z-Stacks of 200 μm depth into the specimens were captured with 2.39 μm intervals between slices. Projections of three-dimension, top view, front view, and side view of maximum projection images were obtained individually.

#### 2.5.3. Osteoblast Differentiation Gene Levels in the MSCs Cultured in the HLC/HLC-BMP Sponges

The expression of osteo-related mRNA transcripts was obtained by RT-qPCR using the same method from our previous study [10]. Briefly, RNA was isolated from MSCs at 3, 5, 7, and 12 days after seeding in each sponge (Cylinder: 34 mm in diameter × 3 mm in height), and their cDNA was synthesized using a Transcriptor First Strand cDNA Synthesis Kit (Roche, Basel, Switzerland). *Runx-2* (runt-related transcription factor 2), *ALP* (alkaline phosphatase) and *OPN* (osteopontin) genes were analyzed. Table 1 lists the primer sequences used. At least three independent experiments were performed.

#### 2.5.4. Osteo-Related Protein Expression in the HLC/HLC-BMP Sponges Cultured with MSCs

Osteo-related protein expression levels in MSCs were then measured using the Western blot method in our previous study [43]. Western blot analysis was carried out for Runx-2, ALP and OPN expression of MSCs which was cultured in each scaffold at days 3, 5, 7, and 12, and β-actin was used as an internal control. The primary antibodies were β-actin (Abcam, ab8227), Runx-2 (Abcam, ab114133), ALP (Abcam, ab83259), and OPN (Novusbio, NB110-89062SS, Centennial, CO, USA). The secondary anti-bodies conjugated with horseradish peroxidase were goat anti-mouse IgG (Peroxidase-conjugated, Jackson, 115-035-003) and goat anti-rabbit IgG (Peroxidase-conjugated, Jackson, 111-035-003).

### 2.6. Ectopic Bone Regeneration of HLC/HLC-BMP Sponges

#### 2.6.1. Animals and Surgery

All procedures of the in vivo assays were approved by the Northwest University IACUC. Eight Kunming mice were prepared (half male and half female, 4 weeks old, weight 22~25 g from the Animal Center, the Fourth Military Medical University, Xi’an, China). The mice were anesthetized with an intraperitoneal injection of 10% chloral hydrate and 1 cm skin incisions were cut in both hind limbs, which were parallel to the longitudinal axis of the muscle fibers, to create an intramuscular pocket to insert the implant. Each mouse received two implants randomly assigned with either HLC or HLC-BMP (*n* = 8 /group, Cylinder: 5 mm in diameter × 3 mm in height). All mice were sacrificed at 4 weeks by CO_2_ inhalation, and the implants were taken for X-rays.

#### 2.6.2. Histology of HLC/HLC-BMP Implant

The method for histology evaluation was according to our previous study [10]. Briefly, the harvested implants were fixed with 4% paraformaldehyde, then demineralized for 5 days in 5% formic acid, followed by dehydrating in alcohol and embedding in paraffin and then cut in the section of 7 μm. H&E (Hematoxylin and eosin), MTS (Masson’s trichrome staining) and Ponceau’s stains on these sections were then used to check new bone formation under a light microscope (VHX-5000, Keyence, Japan).

### 2.7. Rat Cranial Defect Repair Model

#### 2.7.1. Animals and Surgery

Twenty-seven SD rats were used in this study (6~8 weeks old, weight 220~250 g from the Animal Center, the Fourth Military Medical University). The rats were anesthetized with an intraperitoneal injection of 10% chloral hydrate. A calvarial critical size defect was then traumatized with a bone trephine bur for each rat. After the trephined calvarial disk (8 mm in diameter) was removed, the two kinds of scaffolds with cylinder of diameter and height: 8 × 3 mm (HLC group and 1 μg HLC-BMP group, i.e., each piece of HLC loaded with 1 μg rhBMP-2; *n* = 6 for each group) were implanted in the bone defects, those without implants in the bone defects were used as controls (*n* = 6). The skin incisions were then closed with sutures. The rats were put back for normal breeding and sacrificed 2 and 4 weeks after the surgery for micro-computed tomography (Micro-CT, μCT) evaluation and histological examination. To distinguish the effects of the different dose of rhBMP-2 in bone repairing, we further conducted two groups of implanting for 8 weeks using additional three rats each with the HLC-BMP loaded with 1 μg rhBMP-2 and loaded with 5 μg rhBMP-2 separately, and three rats without implant in the bone defects were used as the control.

#### 2.7.2. Micro-CT Evaluation of Cranial Defect Repairing Using HLC/HLC-BMP Sponges

A Micro-CT (Y. Cheetah, YXLON, Hamburg, Germany) was used for morphological and quantitative examination on the specimens at 80 kV and 50 μA. We selected 10 μm for the 3D resolution. 3D images were then reconstructed based on the serial scanned images (VG Studio, v2.2, Volume Graphics Inc., Heidelberg, Germany). The ratio of the bone volume to the total tissue volume was used for calculating the bone volume fraction (BVF).

#### 2.7.3. Histological and Immune-Histological Analysis of Cranial Defect Repairing Using HLC/HLC-BMP Sponges

After 2, 4, and 8 weeks, the implants were harvested separately and fixed with 4% paraformaldehyde. Thereafter, the specimens were demineralized, dehydrated, embedded, sectioned, and stained with MTS and Ponceau’s stain according to the method in our previous study [10,44].

To evaluate the regenerative bone in the defects, immunofluorescence for OPN and Runx-2 was introduced. Based on our previous method [43], tissue sections were immersed in 0.1% Triton-X100 for 10 min, washed with PBS, and pre-incubated with 5% goat serum for 1 h. Then they were incubated with anti-OPN (host: mouse, Novusbio, NB110-89062SS) and anti-Runx-2 (host: rabbit, Abcam, ab114133) antibodies at 4 °C overnight. On the following day, after washing with PBS, the samples were incubated with donkey anti-mouse secondary antibody (Alexa Fluor^®^ 488-conjugated, Jackson, 715-545-151) and donkey anti-rabbit secondary antibody (Alexa Fluor^®^ 594-conjugated, Jackson, 715-585-152) for 1 h, followed by PBS washing, and finally stained with DAPI for 5 min. The fluorescence images were then captured with a confocal laser scanning microscope (FV1000; Olympus Corporation, Tokyo, Japan). Quantitative evaluation of OPN and Runx-2 expression was done by calculating the area of stained cells per middle-power field as previously [45]. In brief, the percentages of OPN and Runx-2 positive areas were obtained by the ratio of IOD (integrated option density, i.e., 6 randomly selected middle-power fields in each animal, n = 18 fields) to selected region area with multiplying factor of 100%.

### 2.8. Statistical Analysis

Statistical analysis was conducted using SPSS software (v20.0, SPSS Inc., Chicago, IL, USA). Data were presented as mean ± SD. *p* < 0.05 were chosen as significant. The significance of differences between groups was assessed using a two-way analysis of variance (ANOVA) with Tukey’s post-hoc analysis.

## 3. Results

### 3.1. Morphology and Mechanical Properties of the HLC/HLC-BMP, and rhBMP-2 Release Kinetics

**Figure 2:**
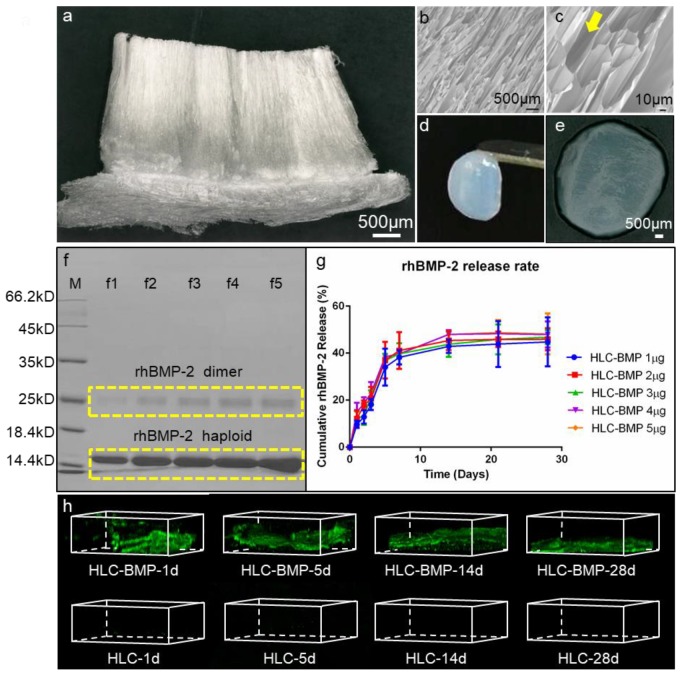
Characterization of the HLC/HLC-BMP sponges. (**a**) Gross view of the lyophilized HLC sponge, by a digital microscope at 30× magnification; (**b**,**c**) SEM micrographs of lyophilized HLC sponge at 100× and 500× magnification, respectively; Yellow arrow indicates the unique vertical pore structure of the HLC sponge; (**d**,**e**) Gross view of the HLC-BMP hydrogel; (**f**) Expression and purification of BMP-2 recombinant human DNA (rhBMP-2) shown by SDS-PAGE. f1–f5 lanes: 1 µg, 2 µg, 3 µg, 4 µg, and 5 µg rhBMP-2 dimer, respectively; (**g**) Cumulative rhBMP-2 release kinetics from the lyophilized HLC-BMP sponges. All HLC-BMP groups were observed with a burst and a 28-day steady release. (**h**) The HLC-BMP hydrogel scaffolds were analyzed using biotinylated anti-human BMP-2 antibody and streptavidin-FITC. The fluorescence images from the stained samples at days 1, 5, 14, and 28, respectively, confirmed that the BMP-2 still remained in the scaffold.

The HLCs were expressed by recombinant *E. coli* BL21. After purification, cross-linked with TG and lyophilized, the HLCs exhibited loose and spongy characteristics (Figure 2a). SEM images (Figure 2b,c) of the HLC sponge showed high porosity, large pore size, and notably a unique vertical pore structure with over 99% porosity (Supplementary Materials Figure S1). After absorbed 200 µL rhBMP-2 solution consisting of 1 µg rhBMP-2 dimer, the HLC sponge changed into HLC-BMP hydrogel (Figure 2d,e).

The cumulative rhBMP-2 release kinetics from HLC-BMP sponges were measured (Figure 2f,g) after the different concentration of rhBMP-2 were absorbed into HLC sponges. The results by SDS-PAGE are shown in Figure 2f. All HLC-BMP sponges/groups showed a controlled burst release of rhBMP-2 within 1 to 3 days (Figure 2g and Supplementary Materials Table S2), and a sustained 28 days steady release for all concentrations (Figure 2g and Supplementary Materials Table S2). To confirm the rest of the rhBMP-2 was still in the HLC-BMP hydrogel scaffold, we analyzed the scaffolds using biotinylated anti-human BMP-2 antibody and streptavidin-FITC. The fluorescence images from the stained samples at days 1, 5, 14, and 28 (Figure 2h) confirmed that the rhBMP-2 was still in the scaffold.

We measured the mechanical properties of HLC without cross-linking, the cross-linked HLC and HLC-BMP sponges. The Young’s modulus of the cross-linked HLC sponge was 2.17 ± 0.43 MPa, which was significantly higher than that of the HLC without cross-linking (1.74 ± 0.06 MPa, *p* < 0.05, n = 4), while Young’s modulus was 2.14 ± 0.61 MPa for the HLC-BMP sponge, which showed similar to the cross-linked HLC sponge (*p* > 0.05, n = 4). The max. compressive forces also showed similar results (*p* > 0.05, n = 4) with 103.23 ± 6.16 N for the HLC sponge, and 102.65 ± 7.98 N for the HLC-BMP sponge; all of them were significantly larger than that of the HLC without cross-linking (21.14 ± 2.80 N, *p* < 0.05, n = 4). Overall, the HLC-BMP sponges appeared to have the same porous micro-structure as the pure HLC sponges, making both sponges suitable for cell residence.

The mechanical properties of the hydrogel are affected by several factors, such as the concentration of HLC used and time of cross-linking with TG. The principle to select for hydrogel-forming is to choose a relative stable strength with fast possible gelation time. Of course, the focus should also be on how the hydrogel would fit when loading with rhBMP-2, for example, in this case. Ten percent (*w*/*v*) HLC/(20 U/g HLC) TG hydrogel was chosen to load rhBMP-2 in this study due to its excellent mechanical properties (stable, strength, no significant difference before/after the loading of rhBMP-2). In our separate experiment, the optimization of the mechanical strength of the hydrogel was thoroughly investigated.

### 3.2. MSCs Attachment, Proliferation and Differentiation in the HLC/HLC-BMP Sponges

**Figure 3:**
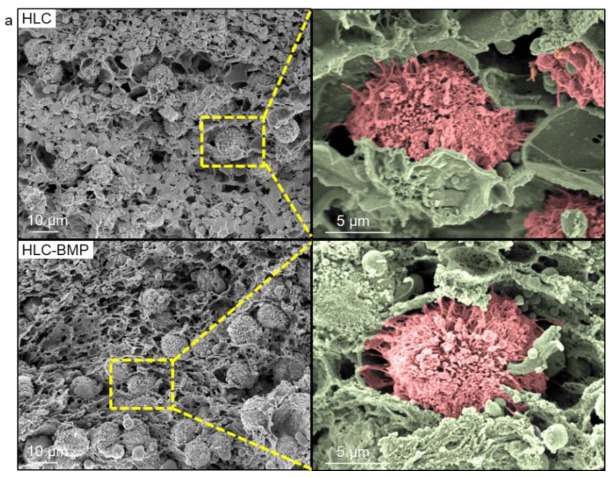

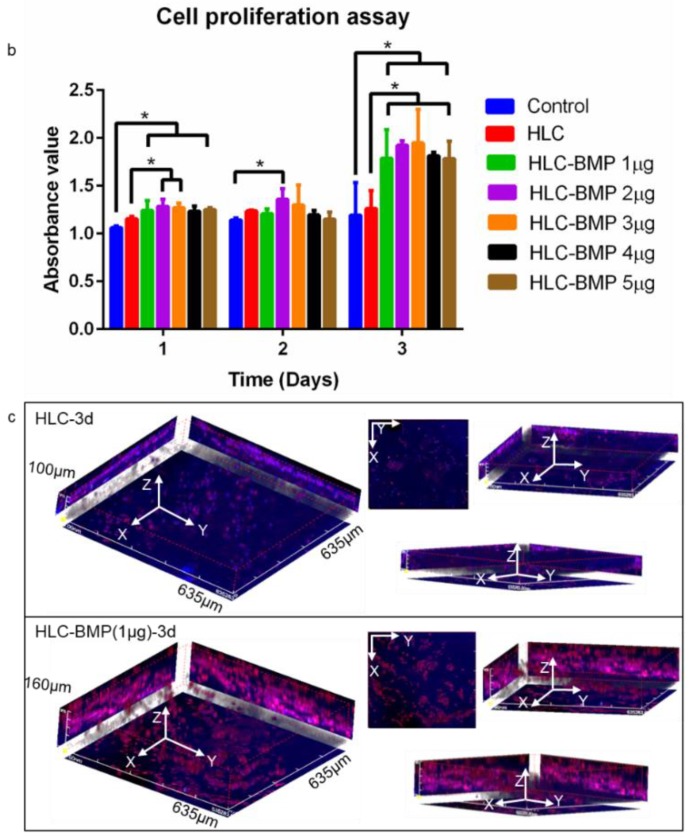
Mesenchymal stem cells (MSCs) attachment and proliferation. (**a**) SEM images of MSCs attached in the HLC and HLC-BMP sponges after culturing for 4 h, respectively. The MSCs in the pores of HLC and HLC-BMP sponges exhibited pseudopodia after only 4 h culture. (**b**) MSCs proliferation on HLC, HLC-BMP (HLC absorbed 1, 2, 3, 4, and 5 µg BMP-2 dimer, respectively) sponges, and culture plate (control) was investigated by Cell Counting Assay Kit-8, respectively. (* p < 0.05, n = 4). (**c**) Confocal 3D reconstructions of encapsulated rat MSCs, stained by a β-actin primary antibody, Alexa Fluor^®^ 594-conjugated donkey anti-rabbit secondary antibody and DAPI, in HLC and HLC-BMP hydrogels.

On times ten to the sixth cells per milliliter MSCs were seeded in each HLC and HLC-BMP sponge. After four hours culturing, the specimens were rinsed, fixed, dehydrated, dried, and gold-palladium coated for SEM observation. The SEM micrographs (Figure 3a) showed that the MSCs were clustered in the pores of HLC and HLC-BMP sponges and exhibited pseudopodia after only 4 h culture. This result indicated that all the sponges presented a porous structure with MSCs adhering well to their pore surface.

MSC proliferation was subsequently checked at days 1, 2, and 3 days after seeding in the HLC/HLC-BMP sponges. Figure 3b showed that the MSCs proliferated well in all specimens. However, cell proliferation in the HLC-BMP sponges was significantly higher compared to that in the HLC sponges and in the cell culture media (control) at day 3 (*p* < 0.05, n = 4).

The cell-sponge complex at day 3 was fixed, stained, and observed by confocal microscopy. The 3D reconstruction of images showed that cells were present in the HLC-BMP hydrogels to a depth of 160 μm along the *Z*-axis (Figure 3c), while cells were only observed to a depth of 100 μm along the *Z*-axis in the HLC hydrogels (Figure 3c). A larger number of cells were also observed in the projection of the XY-plane in the HLC-BMP hydrogels compared to those in the HLC hydrogels, despite the same initial numbers of cells being seeded. Furthermore, red fluorescence (actin) from the HLC-BMP hydrogels was stronger than that obtained from HLC hydrogels, and this indicated that the cells spread and grew better in the HLC-BMP sponges than in the HLC sponges.

**Figure 4:**
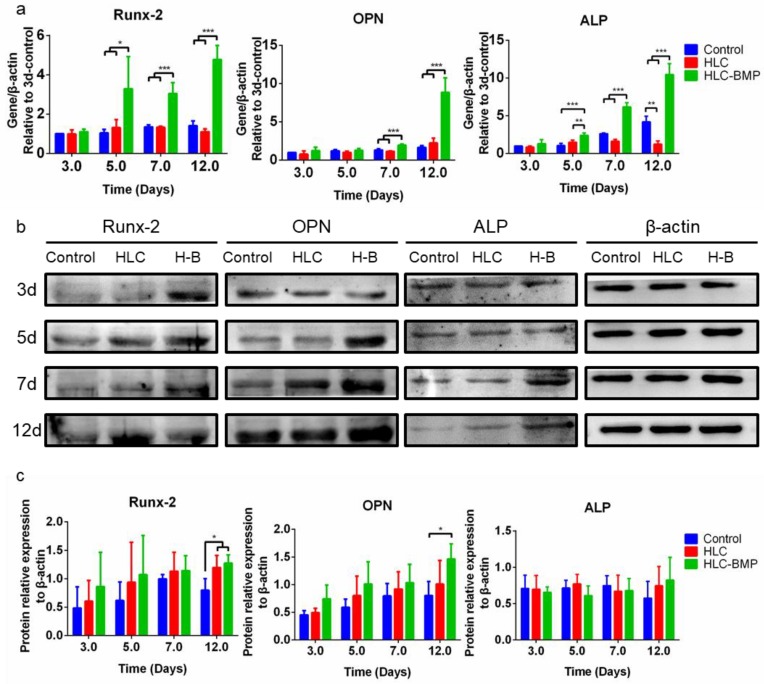
Osteoblast differentiation levels in the MSCs cultured in the HLC/HLC-BMP (1 μg) sponges. (**a**) To explore the mechanisms involved, gene expression levels of osteo-related genes (*Runx-2*, *OPN,* and *ALP*) were assessed by Real-time PCR (* p < 0.05, ** p < 0.01, *** p < 0.001, n = 3). (**b**,**c**) Runx-2, OPN, and ALP protein expression levels were assessed using Western blot (* *p* < 0.05, n = 3).

To confirm that the HLC/HLC-BMP sponges obtained in this study had the ability to promote in vitro osteogenic differentiation, RT-qPCR was performed. Figure 4a shows that *Runx-2*, *OPN,* and *ALP* expression levels in the HLC-BMP sponges were significantly higher than those observed in the HLC sponges and culture plates (control) (*p* < 0.05, n = 3).

Runx-2, OPN, and ALP protein levels were also checked with a Western blot. ALP expressed in MSCs of the HLC-BMP group was slightly higher than that of the HLC and control groups at day 12, but there was no significant difference among the groups (*p* > 0.05, n = 3, Figure 4b,c). The expressions of Runx-2 and OPN proteins in MSCs of the HLC-BMP group at day 12 were significantly higher compared to that of the HLC and control groups (*p* < 0.05, n = 3). Immunoblotting also confirmed that the released rhBMP-2 from HLC-BMP can up-regulate the protein expression level of Runx-2 and OPN and promote MSCs osteoblast differentiation *in vitro*. Here, ALP expression in the HLC-BMP group has not been improved as significantly as for Runx-2 and OPN expressions, although they are all involved in the osteoinduction and bone formation. A possible reason could be the different sensitivities of each protein to this HLC/TG system loading with BMP. Further investigation is needed to explain the role of an individual protein in the bone formation.

### 3.3. Ectopic Bone Formation by HLC/HLC-BMP Sponges

**Figure 5:**
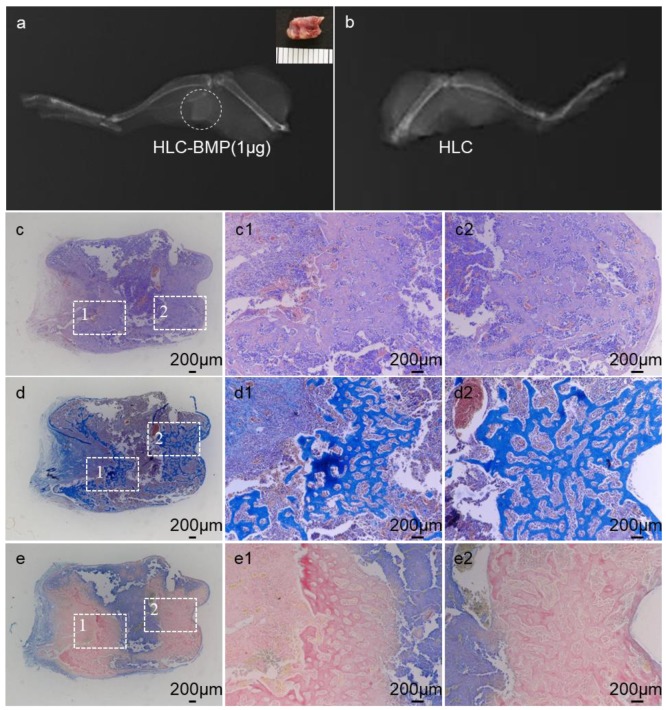
In vivo implantation of HLC-BMP sponges into muscle pouches of the mouse hind limb. (**a**) X-ray micrographs of new bone formation with the use of HLC sponge in 1 µg rhBMP-2 treatment groups at 4 weeks post-implantation. There is nothing formed in the HLC sponge group (**b**). Histological staining of the specimen with (**c**) H&E, (**d**) MTS, and (**e**) Ponceau’s stain, for HLC-BMP after implanted for 4 weeks, respectively.

We used a mouse ectopic bone model to verify the osteoinduction of HLC-BMP sponges. X-rays (Figure 5a) revealed that mineralization with rhBMP-2 at 1 µg dose in the mouse hind limbs, but there is nothing formed in the HLC group (Figure 5b). Newly formed osteoid tissue was visible in the H&E stain of the specimens (Figure 5c). MTS and Ponceau’s stains further revealed that the newly formed tissue was bone. Meanwhile, it revealed abundant collagen deposition in the specimens, indicating abundant ECM formation in the specimens (Figure 5d,e).

### 3.4. Osteogenesis In Situ of HLC-BMP Sponge in Rat Cranial Defect Repair Model

**Figure 6:**
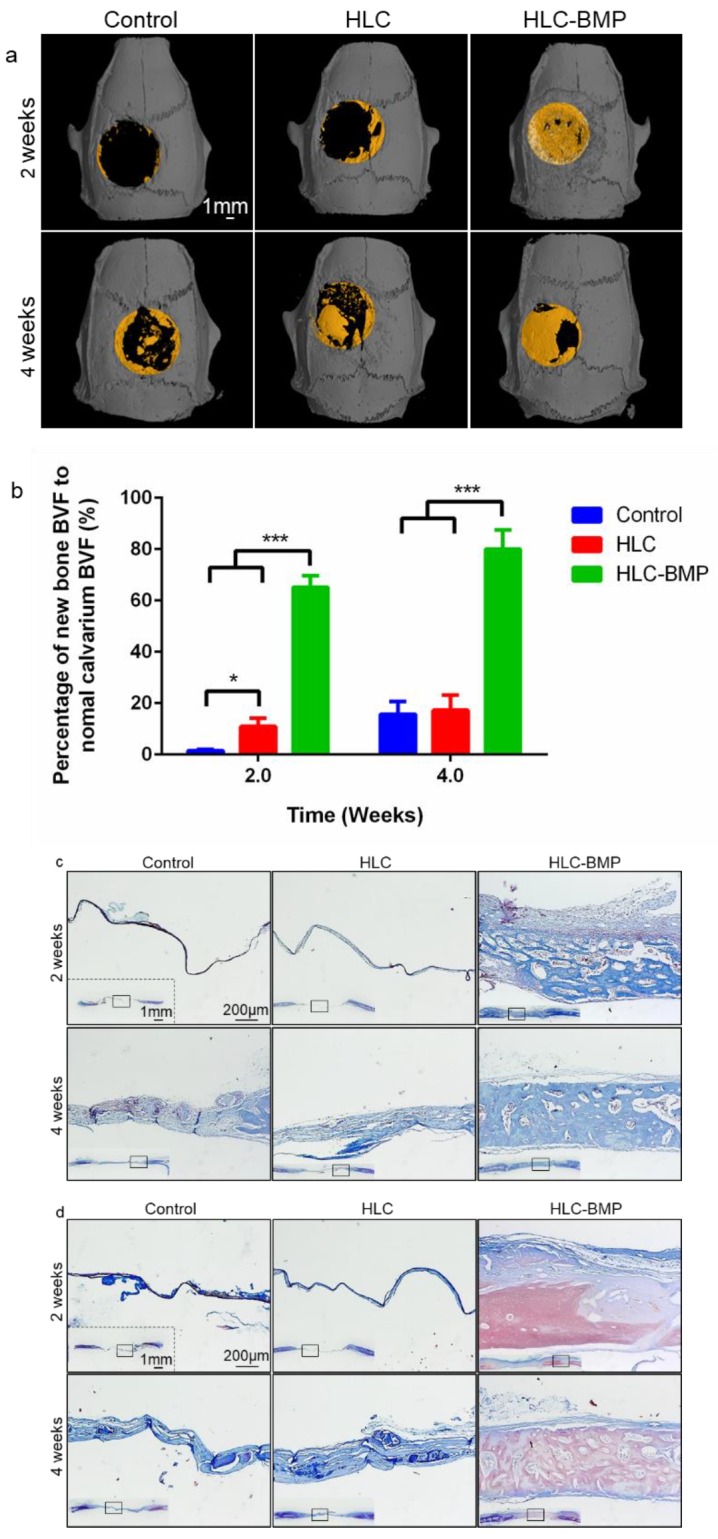
Micro-CT and histological evaluation of the rat cranial defect repair after 2-and 4-weeks implantation. (**a**) Representative micro-CT images of implants (HLC and HLC-BMP) (**b**) Quantitative analysis of bone formation in each group (* p < 0.05, *** p < 0.001, n = 3). Implant histological evaluations on sections stained with (**c**) MTS and (**d**) Ponceau’s stain for the control, HLC and HLC-BMP group, respectively.

We used the rat cranial defect repair model to investigate the effects of the HLC-BMP sponges on osteogenesis in situ and the repair of the rat cranial critical size defect. All rats survived and showed no visible inflammatory reactions, infections, or extrusions. After implanting for 2 weeks, the fresh bone was observed around the central and peripheral part of the calvarial defect in the HLC-BMP group. However, no/few fresh bones were visible around the same place in the control or the HLC group (Figure 6a). After implantation for 4 weeks, only a few bone tissues were observed, and most parts of the defect were still not amended in the control and HLC groups (Figure 6a). In the HLC-BMP group, massive calcified tissues were formed and almost filled the defect site (Figure 6a). The original data of the micro CT images are shown in Figure S2. As shown in Figure 6b, BVF was significantly higher in the HLC-BMP group (2 weeks: 65.21 ± 4.44%; 4 weeks: 79.95 ± 7.57%) than those in the control group (2 weeks: 1.57 ± 0.45%; 4 weeks: 15.67 ± 4.95%) and the HLC group (2 weeks: 10.92 ± 3.28%; 4 weeks: 17.27 ± 5.82%) 2 or 4 weeks after implanting (*p* < 0.001, n = 3, Figure 6b). However, there was no significant difference observed between the HLC group and the control group 4 weeks after implantation (*p* > 0.05, n = 3). These results showed that HLC-BMP implanting could significantly induce bone regeneration in situ and enhance the bone repair percentage within 4 weeks.

After implanting for 2 weeks, a large volume of fresh bone with a little fibrous tissue coating was visible, and the scaffold was partly degraded in the HLC-BMP group (Figure 6c,d). However, few new bones and thin, loose connective tissue were formed in the control and HLC groups (Figure 6c,d). After implanting for 4 weeks, the HLC-BMP group showed more calcified tissue with nearly-complete repair, and the scaffolds were nearly-completely degraded. This contrasts with the limited changes observed in the control and the HLC groups (Figure 6c,d).

**Figure 7:**
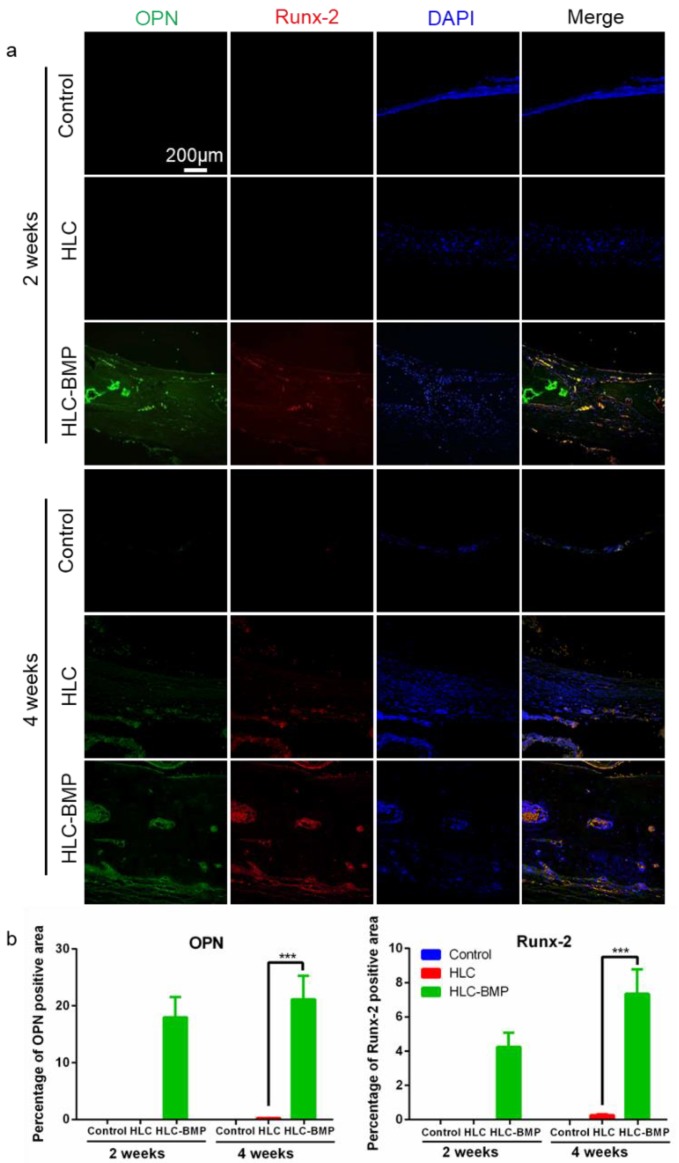
OPN and Runx2 staining of the implants 2 weeks and 4 weeks after implantation. (**a**) Fluorescent microscopy images of OPN and Runx-2 staining implants were recorded in the HLC and the HLC-BMP groups, respectively, at week 2 and 4 post-implantation. Nuclei were stained in blue with DAPI, OPN was stained in green and Runx-2 was stained in red. (**b**) Quantitative estimation of OPN and Runx-2 expression in each group (*** p < 0.001, n = 18).

We further tested immunofluorescence of osteoblast differentiation markers (Preosteoblast marker: Runx-2 [46]; Osteoblast marker: OPN). The HLC-BMP group exhibited a greater intensity of staining for OPN and Runx-2 at each time point, while only faint staining was seen in the HLC group 4 weeks after implantation. No staining was visible in the control group after 2 weeks or 4 weeks implantation (Figure 7a). Immunofluorescence staining of OPN and Runx-2, therefore, revealed that osteo-related proteins showed a significantly greater expression in the HLC-BMP group compared to that in all other groups for each chosen time point (*p* < 0.001, n = 18, Supplementary Materials Table S3 and Figure 7b).

### 3.5. Distinguish the Effects of the Different Dose of BMP-2 Release from the HLC-BMP Sponge in Rat Cranial Defect Repair Model

**Figure 8:**
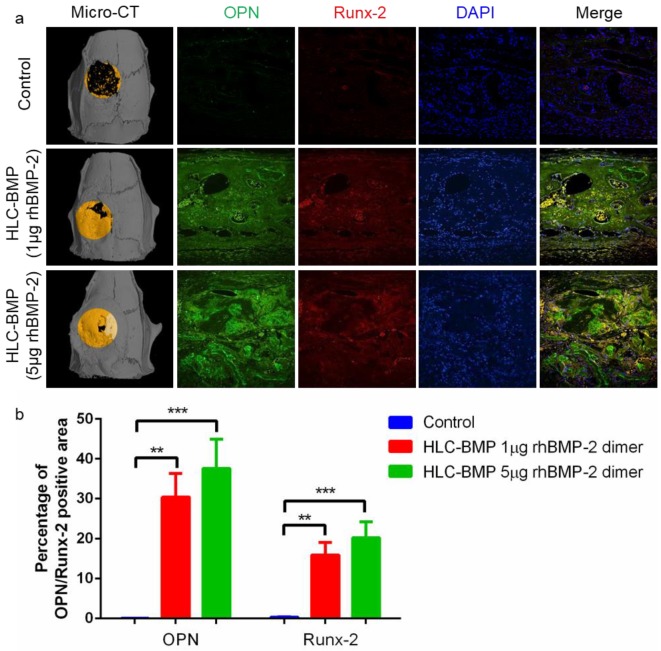
Micro-CT and fluorescent microscopy analysis of the rat cranial defect repair using different doses of BMP-2 at 8 weeks after implantation. (**a**) Representative micro-CT images and immunofluorescent staining of implants (Control, HLC-BMP sponge loading with 1 µg and 5 µg rhBMP-2 dimer, respectively) 8 weeks after implantation. Nuclei were stained blue with DAPI, OPN was stained green, and Runx-2 was stained red. (**b**) Quantitative estimation of OPN and Runx-2 expression in each group (** p < 0.01, *** p < 0.001, n = 18).

We carried out a test lasting for 8 weeks with HLC-BMP implants loaded with either 1 µg or 5 µg rhBMP-2 (1 µg HLC-BMP implant, 5 µg HLC-BMP implant) in the rat cranial defect repair model. Our results showed that 1 µg HLC-BMP implant was effective, and the percentage of the repair was up to 88.13 ± 6.01% (Figure 8a). However, 5 µg HLC-BMP implant introduced serious bone overgrowth (Figure 8a). Meanwhile, quantitative analysis of related gene expression showed that OPN and Runx-2 expressed significantly more in 5 µg HLC-BMP implant group compared to that observed in the control group (n = 18, *p* < 0.001, Figure 8b). The percentage of positive areas for OPN and Runx-2 was 37.55 ± 7.38% and 20.74 ± 3.99%, respectively, in the HLC-BMP-5 µg group, which was also slightly higher than that (OPN: 30.39 ± 5.98%, Runx-2: 15.93 ± 3.13%) observed in the HLC-BMP-1 µg group (Supplementary Materials Table S4). All the above data suggested that although both HLC-BMP groups can maintain osteoinduction and bone formation, only the HLC-BMP-1 µg group was effective in both and had no side effects.

## 4. Discussion

The failure and adverse side effects of BMP-2 on bone repair are mainly caused by the high dose usage and the too fast process of its delivery. Therefore, significant efforts have been made to obtain the “ideal” hydrogel/matrix to load with a low dose BMP-2 and to keep its release sustained, such as a matrix of PEGylated fibrinogen hydrogel [47], fibronectin-like peptide absorbed collagen [12], collagen and hyaluronan [21]. However, the effective and safe dose usage of the BMP-2 has never been seriously considered rather than purely chasing the lowest dose usage and extended-release time. In this study, we used our newly designed and verified HLC and cross-linked it with TG to form a specific hydrogel system, which can improve the BMP-2 utilization rate, and magnify its effective controlled release. Through this sensitive delivery system, the effective dose (1 µg) usage of BMP-2 for bone repair was achieved, and the dose (5 µg) of BMP-2 causing the side effect of overgrowth was distinguished for the first time. In addition, these sensitive and powerful HLCs can be mass-produced (5000 g/500 L fermenter) in our lab. Therefore, the supply shortage of HLC was solved, and enough sensitive and powerful carriers were available to investigate the most appropriate dose of BMP-2 in clinical treatments on various disease states.

It was reported that cells can play to their specific function when they are at the right size within a specific 3D environment [48]. For example, only when the size of the cells is at the most appropriate interval (3500–4500 cubic microns), can the stem cells differentiate into osteoblast [48]. In this study, applying a similar method of “gradient freeze-drying” on the special designed HLC, we were able to obtain a unique pore size and shape of the scaffold (Figure 2b,c) appropriate to support the stem cells differentiation into osteoblast (Figure 4). Meanwhile, with this unique vertical pore structure (Figure 2b,c), the porosity of the HLC (or HLC-BMP) scaffold was over 99% (Supplementary Materials Figure S1), which was higher than that of any previous collagen scaffold [37]. This unique vertical pore structure of HLC sponge was, therefore, able to provide an appropriate space for MSCs to reside, migrate, proliferate, and differentiate (Figure 2b,c, Figure 3; Figure 4). Moreover, the HLC-BMP sponge possessed both a burst and a sustained BMP-2 release profile in vitro (Figure 2g,h, Supplementary Materials Table S2). These two factors together induced extremely effective cell proliferation and differentiation (Figure 3; Figure 4). The optimum burst duration was 1 to 3 days in this study (Supplementary Materials Table S2), and this was good and fast enough to release the BMP-2 in such a short period for the BMP-2 to function. After that, there was a sustained BMP-2 release until 28 days in vitro. We also tested in vivo BMP-2 release. After HLC loading with 1 to 5 μg, BMP-2 was implanted into the rat, the plasma was collected at 1, 2, 3, 5, 7, 14, 21, and 28 days, respectively, and then the BMP-2 was tested using the Human BMP2 ELISA Kit (Human BMP2 ELISA Kit, Abcam, ab119581, Lot GR3174552-1). However, we did not find the BMP-2 in the plasma of the rat. Further on, we tested the BMP-2 in the major organs of the rat using the fluorescently labeled BMP-2. Our results showed that the BMP-2 were either very low or low below detectable levels in the brain, heart, lungs, liver, and spleen (data not shown). We, therefore, concluded that BMP-2 release should be more locally presented. Using in vivo assays, the bone volume (BV) of newly formed bones was evaluated by micro-CT, and the histomorphometric analysis was performed to evaluate the bone volume fraction (BVF). The expression of osteogenic markers, such as OPN, Runx-2, and ALP, was also evaluated by immunofluorescence. Our results showed that the quality of bone formed was satisfactory.

Adjusting the biological template of recombinant HLC, changing the concentration of HLC and cross-link agent (TG), and modifying the vertical pore structure of HLC would further help the interaction between the HLC and the BMP-2 and improve the release profile of the BMP-2. We named this special relationship between HLC and BMP-2 as “dual beneficial interaction”. This “dual beneficial interaction” between the HLC and the BMP-2 resulted in the mechanical strength and the controlled biodegradability (complete biodegradation after new bone formation, as shown in Figure 6). Together with in vivo evaluations of the HLC-BMP sponge’s effectiveness for bone repair (Figure 5, Figure 6 and Figure 7), we provided strong evidence that the carefully designed HLC sponge in this study is suitable for cell infiltration and repair tissue development (Figure 3a). Meanwhile, we compared our HLC sponge with the commercial collagen sponge (Supplementary Materials Table S5) and found that the designed HLC sponge in our study can release the BMP-2 significantly more effectively than the commercial bovine Achilles tendon-extracted collagen sponge does [21]. Using the similar cranial defect repair model and loading with the same dose of BMP-2, the repair rate of human-like collagen sponge was also significantly higher than that of commercial bovine skin-extracted collagen sponge [25]. Therefore, the novel HLC sponge in this study created a template for “dual beneficial interaction” with BMP-2 or other growth factors.

Due to its strong binding affinity, BMP-2 possesses the ability to reduce its release rate and maintain the low dose usage for the implants [10] and thus promote its safety within the implants [49,50]. In recent years, many studies have tried to control the dose usage through chemical conjugation of BMP-2 [51,52,53]. However, either high concentrations of the BMP-2 solution were still used (>1 mg/mL) [51,52] or there was a lack of in vivo evidence showing that the successful immobilization of BMP-2 on the surface of their scaffolds was achieved at lower concentrations (100 or 500 ng/mL) [53]. In our study, without chemical conjugation of BMP-2, the HLC sponge can load with a trace amount BMP-2 (~1 µg BMP-2 dimer) with a controlled burst within 1 to 3 days and a sustained release over 28 days to promote the effective use of the implants (Figure 2f–h, 6) and showed no side effects. However, 5 µg HLC-BMP implant introduced serious bone overgrowth (Figure 8a). Observation of this interesting phenomenon indicated that the HLC in our study seems to have an ability to “distinguish” the tiny changes in a dose of BMP-2 used for maintaining osteoinduction and bone formation and expose any side effect it may bring. This showed that the novel HLC and HLC-BMP sponges created in our study provided a unique platform to verify the safe dose usage of BMP-2. Testing a wider range of BMP-2 dose usage would further illustrate the safe guide on using BMP-2. This is particularly important and beneficial for the clinical application.

## 5. Conclusions

In this study, we created a novel recombinant HLC and cross-linked the HLC with TG to form an HLC sponge. This HLC sponge has strong mechanical strength, controlled biodegradability, unique vertical pore structure, and can magnify and distinguish the tiny changes of a dose of BMP-2 used for maintaining osteoinduction and bone formation. The mouse ectopic bone model and the rat cranial defect repair model employed demonstrated that the HLC-BMP loaded with BMP-2 (l µg) can rapidly and effectively repair the bone defect nearly completely. However, the HLC-BMP loaded with a slightly higher dose of BMP-2 (5 µg) can introduce serious bone overgrowth. This showed that the novel HLC sponges created in our study can be used to verify the BMP-2 safe dose usage in bone repair. Furthermore, the gene template of the HLCs can be customized, and the HLCs with a high-yield product of 5000 g/500 L fermenter can be obtained through low-cost fermentation. This can solve the supply shortage of carrier protein. All our results showed that our novel HLC could be developed into a customizable delivery system for other growth factors and will be extremely valuable for any defect repair and tissue regeneration in both research and clinical application.

## Figures and Tables

**Table 1:** Details of primers sequence for RT-qPCR.

Gene	Primers	Product Size (bp)
*Runx-2*	5′-GCCACTTACCACAGAGCTATTA-3′(F)	106
	5′-GGCGGTCAGAGAACAAACTA-3′(R)	
*OPN*	5′-AGGAGTTTCCCTGTTTCTGATG-3′(F)	110
	5′-GCAACTGGGATGACCTTGATA-3′(R)	
*ALP*	5′-ACAAGTGTGGCAGTGGTATT-3′(F)	104
	5′-CTGCTTGAGGTTGAGGTTACA-3′(R)	
*β-actin*	5′-CTGTGCTATGTTGCCCTAGAC-3′(F)	115
	5′-GCTCATTGCCGATAGTGATGA-3′(R)	

## Supplementary Materials

The following are available online at https://www.mdpi.com/2218-273X/9/9/450/s1, Figure S1: Mercury porosimetry report of the HLC scaffold, Figure S2: Micro-CT images of the rat cranial defect repair, Table S1: Expression of the recombinant human-like collagens, Table S2: Original data of rhBMP-2 Release Kinetics, Table S3: Original data of quantitative estimation of OPN and Runx-2 expression in each implant 2 weeks and 4 weeks after implantation, Table S4: Original data of quantitative estimation of OPN and Runx-2 expression in each implant 8 weeks after implantation, Table S5: Comparison the commercial collagen sponge and the recombinant human-like collagen sponge.
