# Supplementary material for: Newly Designed Human-Like Collagen to Maximize Sensitive Release of BMP-2 for Remarkable Repairing of Bone Defects
Source: Biomolecules. 2019 Sep 4;9(9):450. doi: 10.3390/biom9090450 (PMC6769454; doi:10.3390/biom9090450)
Supplement: Supplementary file 1 [file biomolecules-09-00450-s001.pdf]

Supplementary Materials

Article

Newly Designed Human-like Collagen to Maximize Sensitive Release of BMP-2 for Markedly Repairing of Bone Defect

Zhuoyue Chen[1,2], Zhen Zhang[1], Xiaoxuan Ma[2], Zhiguang Duan[2], Junfeng Hui[2], Chenhui Zhu[2], Donggang Zhang[3], Daidi Fan[2*], Lijun Shang[4*], Fulin Chen[1*]

[1]Provincial Key Laboratory of Biotechnology of Shaanxi; Key Laboratory of Resource Biology and Modern Biotechnology in Western China, Ministry of Education; Northwest University, 229 TaiBai North Road, Xi'an, Shaanxi Province 710069, P.R. China;

[2]Shaanxi Key Laboratory of Degradable Biomedical Materials; Shaanxi R&D Center of Biomaterial and Fermentation Engineering; School of Chemical Engineering, Northwest University, 229 TaiBai North Road, Xi'an, Shaanxi Province 710069, P.R. China;

[3]Yantai Zhenghai Biotechnology Co, Ltd., 10 Hengshan Road, Yantai Economic & Technological Development Area, Yantai, Shandong Province 264000, P.R. China;

[4]School of Chemistry and Biosciences, Faculty of Life Sciences, University of Bradford, Bradford, BD7 1DP, UK;

***Correspondence:** fandaidi@nwu.edu.cn; +86-29-88302632 (D. D. Fan); l.shang1@bradford.ac.uk; +44-1274-234676 (L. Shang); chenfl@nwu.edu.cn; 86029-88302411 (F. L. Chen)

Figure S1. Mercury porosimetry report of the HLC scaffold The porosity of the HLC scaffold was over 99%

Figure S2. Micro-CT images of the rat cranial defect repair Micro-CT images of control, HLC and HLC-BMP implants groups

Table S1. Expression of the recombinant human-like collagens

Table S2. Original data of rhBMP-2 Release Kinetics.

Table S3. Original data of quantitative estimation of OPN and Runx-2 expression in each implant 2 weeks and 4 weeks after implantation.

Table S4. Original data of quantitative estimation of OPN and Runx-2 expression in each implant 8 weeks after implantation.

Table S5. Comparison the commercial collagen sponge and the recombinant human-like collagen sponge

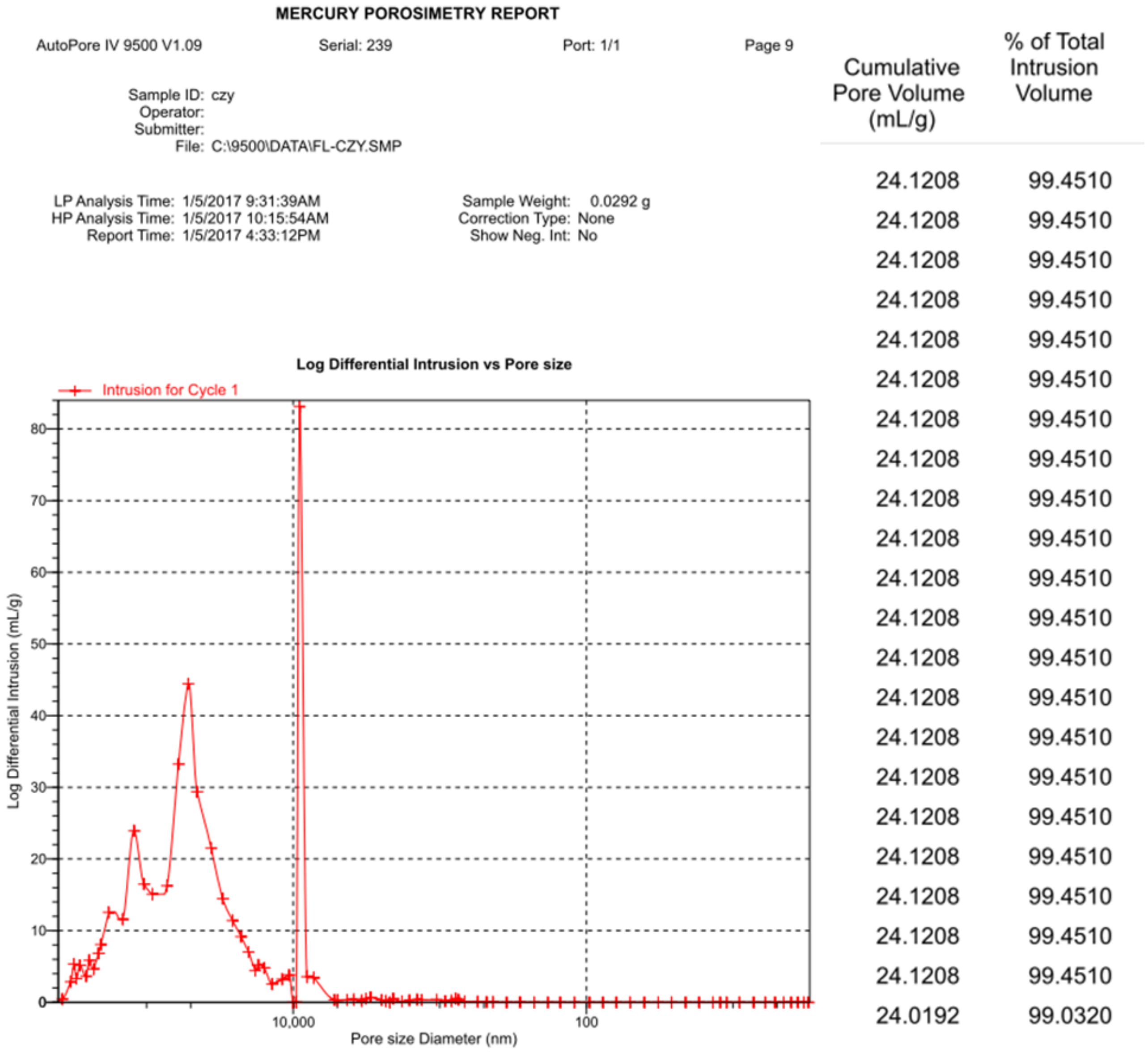

Cumulative Pore Volume (mL/g)	% of Total Intrusion Volume
24.1208	99.4510
24.1208	99.4510
24.1208	99.4510
24.1208	99.4510
24.1208	99.4510
24.1208	99.4510
24.1208	99.4510
24.1208	99.4510
24.1208	99.4510
24.1208	99.4510
24.1208	99.4510
24.1208	99.4510
24.1208	99.4510
24.1208	99.4510
24.1208	99.4510
24.1208	99.4510
24.1208	99.4510
24.1208	99.4510
24.1208	99.4510
24.1208	99.4510
24.1208	99.4510
24.0192	99.0320

Figure S1. Mercury porosimetry report of HLC scaffold. The porosity of the HLC scaffold was over 99%.

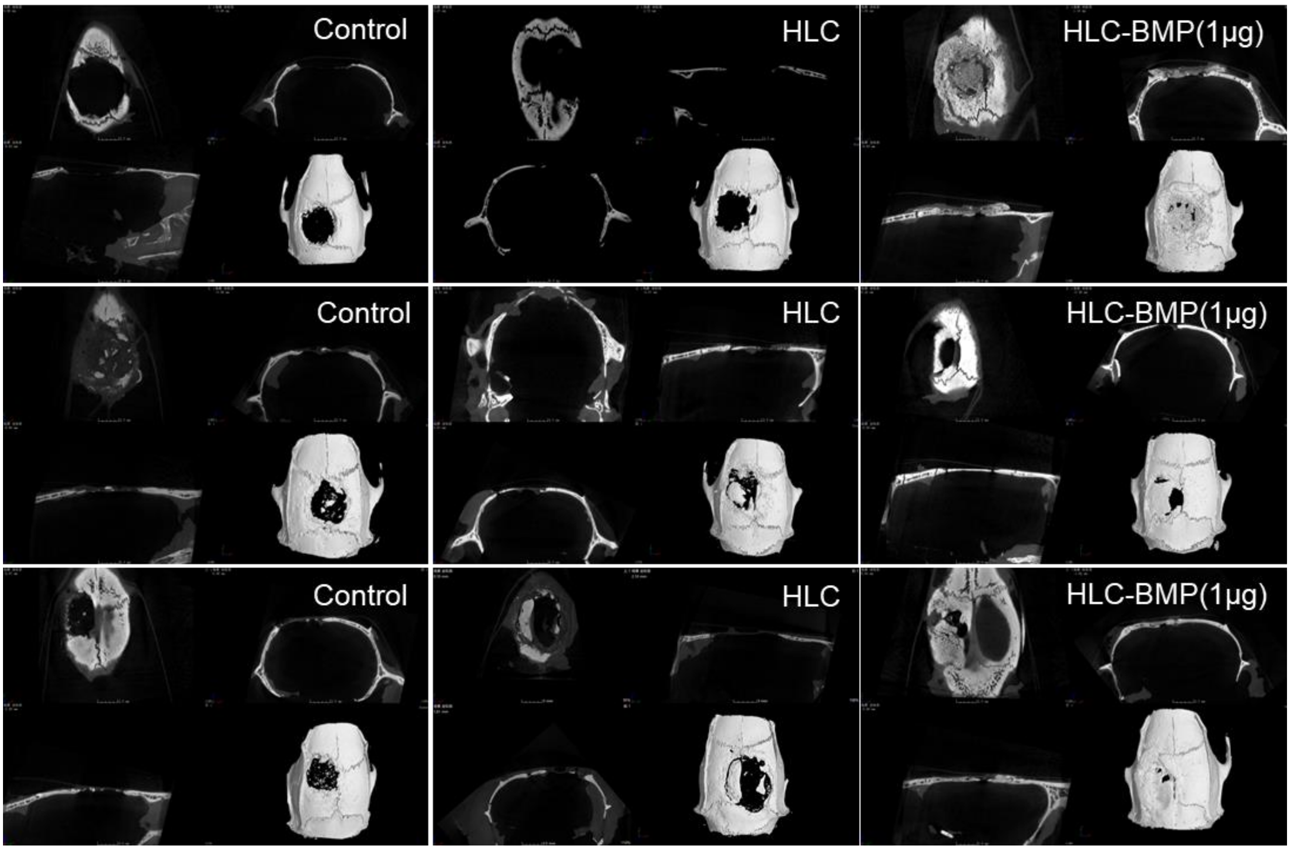

Figure S2. Micro-CT images of the rat cranial defect repair Micro-CT images of control, HLC and HLC-BMP implants groups

Table S1. Expression of the recombinant human-like collagens

Host	Yield	Molecular Wt.	Ref.
E. coli origami (DE3)	260 mg/L in a 10 L fermentor	35 kDa	Tang *et al.* [1]
E. coli Rosetta (DE3)	Shake flask culture	40 kDa	Yang *et al.*[2]
E. coli BL21 (DE3)	14 g/L in a 500 L fermentor	93 kDa	Fan *et al.* [3, 4]

(1). Tang, Y.; Yang, X.; Hang, B.; Li, J.; Huang, L.; Huang, F.; Xu, Z. Efficient Production of Hydroxylated Human-Like Collagen Via the Co-Expression of Three Key Genes in Escherichia coli Origami (DE3). Applied Biochemistry & Biotechnology 2016, 178, 1458-1470.

(2). Yang, J.; Jie-Ying, Y. U.; Wang, M.; Shao-Min, W. U.; Yi, M. A.; Wang, J. F. Expression, Purification, and Functional Characterization of Recombinant Human-Like Type i Collagen Peptide in E.coli. Modern Food Science & Technology 2016.

(3). Fan, D. D.; Luo, Y.; Mi, Y.; Ma, X. X.; Shang, L. Characteristics of Fed-Batch Cultures of Recombinant Escherichia Coli Containing Human-Like Collagen cDNA at Different Specific Growth Rates. Biotechnology Letters 2005, 27, 865-870.

(4). Zhu, C.; Fan, D.; Duan, Z.; Xue, W.; Shang, L.; Chen, F.; Luo, Y. Initial Investigation of Novel Human-Like Collagen/Chitosan Scaffold for Vascular Tissue Engineering. Journal of Biomedical Materials Research Part a 2010, 89A, 829-840.

Table S2. Original data of rhBMP-2 Release Kinetics. Day 1~3: Burst release period; Day 3~28 and later: Sustained release period. The percentage of rhBMP-2 release is no difference among loading with different dose rhBMP-2 groups at each time point ($p > 0.05$, n=4).

**Time (Days)**	**HLC-BMP 1μg (%)**				**Average**	**STD**
1	11.75	9.47	7.96	9.78	9.74	1.56
2	13.60	8.66	14.75	14.47	12.87	2.85
3	16.80	19.57	15.58	20.59	18.13	2.34
5	44.77	26.78	34.68	29.88	34.03	7.86
7	35.76	36.80	37.60	42.69	38.21	3.08
14	43.57	40.70	40.68	46.59	42.88	2.82
21	32.85	44.46	56.54	41.73	43.89	9.78
28	40.79	45.69	58.69	33.99	44.79	10.43
**Time (Days)**	**HLC-BMP 2μg (%)**				**Average**	**STD**
1	15.65	8.57	14.48	9.64	12.09	3.50
2	19.69	15.77	19.58	16.76	17.95	1.99
3	17.54	15.58	23.77	25.80	20.67	4.89
5	46.67	29.68	31.70	39.81	36.96	7.81
7	51.57	39.55	40.76	32.68	41.14	7.81
14	41.80	46.64	42.55	50.57	45.39	4.05
21	45.66	50.46	42.67	44.65	45.86	3.31
28	50.43	43.90	49.12	40.32	45.94	4.69
**Time (Days)**	**HLC-BMP 3μg (%)**				**Average**	**STD**
1	9.56	8.65	13.77	15.86	11.96	3.43
2	17.85	15.80	10.57	9.68	13.47	3.97
3	23.90	22.75	21.85	17.70	21.55	2.70
5	34.55	36.80	35.46	40.70	36.88	2.71
7	45.60	38.59	34.79	39.90	39.72	4.48
14	45.89	36.78	49.59	42.77	43.76	5.42
21	45.85	40.78	54.86	42.09	45.90	6.35
28	43.87	50.45	44.86	47.98	46.79	3.00
**Time (Days)**	**HLC-BMP 4μg (%)**				**Average**	**STD**
1	16.58	18.59	9.48	15.47	15.03	3.92
2	16.69	17.69	22.46	17.58	18.61	2.61

3	27.54	18.40	26.50	19.70	23.04	4.65
5	39.35	36.65	39.33	38.54	38.47	1.27
7	42.37	38.16	38.50	39.59	39.66	1.91
14	45.48	49.84	48.27	48.37	47.99	1.82
21	47.80	47.80	49.77	47.68	48.26	1.01
28	51.69	49.78	50.59	39.70	47.94	5.55
**Time (Days)**	**HLC-BMP 5μg (%)**				**Average**	**STD**
1	16.58	18.59	9.48	15.47	15.03	3.92
2	16.69	17.69	22.46	17.58	18.61	2.61
3	27.54	18.40	26.50	19.70	23.04	4.65
5	39.35	36.65	39.33	38.54	38.47	1.27
7	42.37	38.16	38.50	39.59	39.66	1.91
14	45.48	49.84	48.27	48.37	47.99	1.82
21	45.80	55.80	49.77	43.68	48.76	5.33
28	59.69	39.78	43.59	49.70	48.19	8.69

Table S3. Original data of quantitative estimation of OPN and Runx-2 expression in each implant 2 weeks and 4 weeks after implantation.

**Time (weeks)/group**		**OPN (%)**			**Average**	**STD**
2	Control	0.00	0.00	0.00	0.00	0.00
	HLC	0.00	0.00	0.00	0.00	0.00
	HLC-BMP	21.07	14.13	18.77	17.99	3.54
4	Control	0.00	0.00	0.00	0.00	0.00
	HLC	0.26	0.18	0.24	0.23	0.04
	HLC-BMP	24.77	16.61	22.07	21.15	4.16
**Time (weeks)/group**		**Runx-2 (%)**			**Average**	**STD**
2	Control	0.00	0.00	0.00	0.00	0.00
	HLC	0.00	0.00	0.00	0.00	0.00
	HLC-BMP	4.97	3.33	4.43	4.25	0.83
4	Control	0.00	0.00	0.00	0.00	0.00
	HLC	0.31	0.21	0.27	0.26	0.05
	HLC-BMP	8.60	5.77	7.67	7.35	1.44

Table S4. Original data of quantitative estimation of OPN and Runx-2 expression in each implant 8 weeks after implantation.

**Group**	**OPN (%)**			**Average**	**STD**
Control	0.09	0.06	0.08	0.08	0.02
HLC- 1μg BMP-2 dimer	35.60	23.87	31.72	30.39	5.98
HLC- 5μg BMP-2 dimer	43.98	29.48	39.18	37.55	7.38
**Group**	**Runx-2 (%)**			**Average**	**STD**
Control	0.43	0.29	0.38	0.36	0.07
HLC- 1μg BMP-2 dimer	18.66	12.51	16.62	15.93	3.13
HLC- 5μg BMP-2 dimer	23.75	15.92	21.16	20.27	3.99

Table S5. Comparison the commercial collagen sponge and the recombinant human-like collagen sponge

Source	Company	Release rate	Effect
bovine achilles tendon -extracted collagen[5]	Helistat®, Integra Life Sciences Corporation , USA	5 μg BMP-2 dose group released 55.46 ± 2.01% within 1 hour, 84.94 ± 8.68% within 1 day, and 100% at day 2. The 1 μg BMP-2 group released 34.73 ± 8.25% within 6 hours, and 45% at the third day.	The collagen sponge containing 1 μg BMP-2 formed the new bone volume was 0.6 ± 0.3 mm^3 at 8 weeks post-implantation into the rat muscle bag.
recombinant from *E. coli*	Our lab.	HLC containing 5 μg BMP-2 gel released 15.03 ± 3.92% of BMP-2 at day 1, 18.61 ± 2.61% at day 2 and 48.19 ± 8.69% at day 28. The HLC containing 1 μg BMP-2 released 9.74 ± 1.56% at day 1, 18.13 ± 2.34% at day 3, and 44.79 ± 10.43% at day 28.	Using the similar rat cranial defect repair model, the repair rate is 65.21 ± 4.44% at 2 weeks postoperatively. At 4 weeks postoperatively, cranial defect repair rate of the designed HLC loaded with 1 μg BMP-2 group achieved 79.95 ± 7.57%.
bovine skin -extracted collagen[6]	I-PC, Koken Co. Ltd. Tokyo, Japan	Collagen gel containing 60 μg of BMP-2 released 6% of BMP-2 at day 1. About 33% of the total BMP-2 was released from the collagen gel within 15 days.	None of the new bone tissue appeared in the mouse cranial defect area at 2 weeks postoperatively. At 4 weeks postoperatively, the cranial defect repair rate of the commercial sponge loaded with almost 1 μg BMP-2 group was 40.24 ± 9.70%.

(5). Bhakta, G.; Lim, Z. X. H.; Rai, B.; Lin, T.; Hui, J. H.; Prestwich, G. D.; Wijnen, A. J. V.; Nurcombe, V.; Cool, S. M. The Influence of Collagen and Hyaluronan Matrices On the Delivery and Bioactivity of Bone Morphogenetic Protein-2 and Ectopic Bone Formation. Acta Biomaterialia 2013, 9, 9098-9106.

(6). Zhao, J.; Shinkai, M.; Takezawa, T.; Ohba, S.; Chung, U.; Nagamune, T. Bone Regeneration Using Collagen Type I Vitrigel with Bone Morphogenetic Protein-2. Journal of Bioscience and Bioengineering 2009, 107, 318-323.
